# Microbial Contributions for Rice Production: From Conventional Crop Management to the Use of ‘Omics’ Technologies

**DOI:** 10.3390/ijms23020737

**Published:** 2022-01-10

**Authors:** Febri Doni, Nurul Shamsinah Mohd Suhaimi, Muhamad Shakirin Mispan, F Fathurrahman, Betty Mayawatie Marzuki, Joko Kusmoro, Norman Uphoff

**Affiliations:** 1 Department of Biology, Faculty of Mathematics and Natural Sciences, Universitas Padjadjaran, Jatinangor 45363, West Java, Indonesia; betty@unpad.ac.id (B.M.M.); joko.kusmoro@unpad.ac.id (J.K.); 2 Institute of Biological Sciences, Faculty of Science, University of Malaya, Kuala Lumpur 50603, Malaysia; nurull@um.edu.my (N.S.M.S.); shakirin@um.edu.my (M.S.M.); 3 Centre for Research in Biotechnology for Agriculture (CEBAR), University of Malaya, Kuala Lumpur 50603, Malaysia; 4 Department of Agrotechnology, Faculty of Agriculture, Universitas Islam Riau, Pekanbaru 28284, Indonesia; fathur@agr.uir.ac.id; 5 SRI International Network and Resources Center, Cornell University, Ithaca, NY 14853, USA; ntu1@cornell.edu

**Keywords:** rice, microbiome, plant–microbe interactions, symbiosis, sustainable agriculture

## Abstract

Rice, the main staple food for about half of the world’s population, has had the growth of its production stagnate in the last two decades. One of the ways to further improve rice production is to enhance the associations between rice plants and the microbiome that exists around, on, and inside the plant. This article reviews recent developments in understanding how microorganisms exert positive influences on plant growth, production, and health, focusing particularly on rice. A variety of microbial species and taxa reside in the rhizosphere and the phyllosphere of plants and also have multiple roles as symbiotic endophytes while living within plant tissues and even cells. They alter the morphology of host plants, enhance their growth, health, and yield, and reduce their vulnerability to biotic and abiotic stresses. The findings of both agronomic and molecular analysis show ways in which microorganisms regulate the growth, physiological traits, and molecular signaling within rice plants. However, many significant scientific questions remain to be resolved. Advancements in high-throughput multi-omics technologies can be used to elucidate mechanisms involved in microbial–rice plant associations. Prospectively, the use of microbial inoculants and associated approaches offers some new, cost-effective, and more eco-friendly practices for increasing rice production.

## 1. Introduction

Rice is one of the most widely consumed grains, and it is a staple food for more than 3.5 billion of the world’s population. The current global paddy production is reported to be around 755 million tonnes, with a global cultivated area of rice around 167 million hectares. The demand for rice will surely continue to increase due to the steady growth of the world’s population [1]. For some five decades starting in the 1960s, rice yields around the world were improved substantially by the use of high-yielding varieties, pesticides, fertilizers, and improved irrigation systems [2,3]. 

However, in the past two decades, increases in rice yield and cultivation area have stagnated due to limited water for irrigation, increases in the costs of cultivation, and degradation of water and soil quality [4,5,6]. In addition, with greater ecological awareness and concerns for the quality of food consumed, consumers have begun to question the use of inorganic nutrients and crop protection [7,8].

Thus, the development and use of microbial inoculants to stimulate plant growth and health is a propitious option for meeting the expectations of society for more food and a more healthful and sustainable food supply [9,10]. Plant-beneficial associations with microorganisms are commonplace in nature and have been important for plant survival and evolution over many millennia [11,12,13]. The complex, structured, and interconnected microbial networks that exist in and around plants consist of many different taxa with complementary roles. Keystone species have been identified that are crucial for plant health and ecosystem functioning so that underground ecosystems parallel the ecological complexity and interdependence found above-ground [14,15,16].

A multitude of microorganisms stimulate plant growth and productivity through a variety of mechanisms that include improved nutrient acquisition, altered gene expression, enhanced physiological and biochemical traits, and inhibition of phytopathogens [17,18,19]. The purposeful utilization of microorganisms’ capabilities may become particularly important within sustainable, low-input agricultural cropping systems that rely on biological processes rather than on agrochemicals to maintain soil fertility and protect plant health [20,21,22].

Plant microbiomes are reported to be key factors in maintaining soil quality and rice production, being important influences on the growth of plants from seed to maturity [23,24]. This highly distinct group of microorganisms can also have profound impacts in stimulating rice plant resistance to disease and abiotic stresses [25]. Since the 1970s, various studies on rice plant–microbe interactions have shown that harnessing the potential of microorganisms can contribute to sustainable rice production [26,27].

In the past few years, the advancement in high-throughput multi-omics techniques has allowed researchers to study more comprehensively the physiological, biochemical, and molecular mechanisms that are attributable to the plant microbiome for enhancing and regulating the growth of rice. This article reviews current understandings of the roles that plant microbiomes play in enhancing the of growth, production, and health of this essential crop.

This article also discusses the physiological, biochemical, and molecular mechanisms that underlie the interaction between microbes and rice plants in the context of multi-omics approaches (proteomics, transcriptomics, metagenomics, and metabolomics). Better understanding of microbe–rice plant interactions and of the underlying mechanisms involved will be essential for the utilization of these microorganisms by farmers and agricultural practitioners to meet the ever-increasing demand for sustainable food production.

## 2. The Diverse and Dynamic Structures of Rice Microbiomes

Rice fields harbor a bewildering diversity of soil microbes and soil fauna—including nitrogen fixers, nitrifiers, methanogens, plant-growth regulators, methane oxidizers, phosphate-dissolving microbes, and sulfur oxidizers along with decomposers and nutrient recyclers [28,29,30]. The composition of species will vary according to chemical, physical, climatic, and other conditions. For example, in comparison with other soil habitats, rice soils have a predominance of actinomycetes and Gram-positive bacteria. The microbial communities found in floodwater have a majority of Gram-negative bacteria plus algae, while in percolating water, only Gram-negative bacteria are common without algae [31,32,33].

Rice–soil microbial communities are composed of huge numbers of bacterial and fungal species that perform a multiplicity of ecological functions [34]. Various species of archaea, oomycetes, and other microorganisms play salient roles in driving multiple ecosystem functions and ecological processes that maintain soil health and productivity [35,36,37]. However, bacteria and fungi have the leading roles. Bacterial communities are usually dominated by the Proteobacteria, Chloroflexi, Actinobacteria, and Acidobacteria, while the most prominent members in fungal communities are Ascomycota, Basidiomycota, and Glomeromycota [38,39,40].

The microbial communities found in rice field soils are shaped by biotic and abiotic factors such as temperature, precipitation, humidity, pH, agrochemical applications, balance of cations and anions, soil texture, and the rice cultivar planted [23]. Agricultural management practices such as method of crop establishment (transplanting or direct-seeding) and the duration of drying/flooding stages during crop growth also influence the microbial community structure [34,41].

For example, Klinnawee et al. [42] have documented that under water-saturated soil conditions, the symbiotic relationship between mycorrhizal fungi and lowland (flooded) rice plant roots is diminished because, as aerobic microbes, these organisms similar to other fungi are greatly affected by the amount of water in the soil, which determines the availability of oxygen. ITS2 sequencing analysis has shown that keeping rice paddies inundated inhibits the extent of fungal communities in the soil and reduces the abundance of mycorrhizal fungi in rice roots [42].

### 2.1. The Rhizosphere

Compared to archaea and fungi, bacteria are the predominant group found in the rhizosphere soil that surrounds plant roots; and among the bacterial phyla, Proteobacteria is the major phylum found in most rice rhizospheres [43]. Studies in China have found that the most abundant bacterial genera associated with the core microbiota of rice rhizospheres under standard crop management there are *Anaeromyxobacter*, *Arenimonas*, *Arthrobacter*, *Bacillus*, *Bellilinea*, and 15 other genera [44], all of which are known to contribute to the growth and health of rice plants [23].

The microbial communities in the root microbiome of rice are influenced by cultivation practices. For example, organically cultivated soils are enriched with certain genera that have potential for promoting plant growth such as *Anabaena*, *Azospirilllum,* and *Rhodobacter* [45]. Soils on rice farms that are continuously flooded harbor large numbers of methanogenic archaea that generate the greenhouse gas methane. Conversely, in rice-paddy soils that are kept mostly aerobic, methanotrophic bacteria are more dominant. They consume methane for their own needs, keeping this gas from entering the atmosphere [46].

Plant roots exude a wide range of compounds into their surrounding rhizosphere such as sugars, polysaccharides, amino acids, aromatic acids, aliphatic acids, and fatty acids that attract microorganisms to form a mutualistic association. The composition and pattern of root exudates definitely will affect the makeup of microbial communities in the rhizosphere [47,48].

The domestication of rice, both its origins and species, has been influenced by the composition of fungal communities in the rhizosphere. For example, it has been found that five genera of arbuscular mycorrhizal fungi (AMF) (*Acaulospora*, *Claroideoglomus*, *Pacispora*, *Redeckera*, and *Scutellospora*) are significantly correlated with other fungi in the rhizosphere of wild rice, whereas only three groups of AMF, a somewhat different set (*Claroideoglomus*, *Gigaspora*, and *Redeckera*), are significantly correlated with the other fungi that exist in the rhizosphere of domesticated rice [49].

Arbuscular mycorrhizal fungi are also affected by cultivation practices. When the AMF communities inhabiting rice roots growing under the System of Rice Intensification (SRI) were compared with those found under conventional methods of cultivation, all the AMF in the roots sampled from conventional plots belonged to just one genus, *Glomus*, while in the roots of rice plants being grown in an SRI environment, there were gene sequences for both *Acaulospora* and *Glomus* [50].

**Figure 1:**
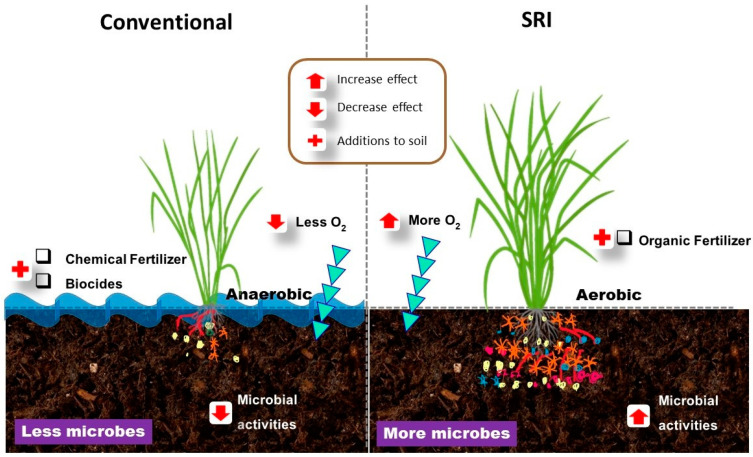
SRI methods produce more robust plant growth and health (**right**) than conventional grown plant (**left**). This is due to the SRI agroecosystems providing a more supportive environment for microbes to grow and to benefit rice plants, while conventional methods limit microbes’ growth and inhibit their effects. If this model is transposed to the larger scale of an SRI rice field, it is understandable why SRI plants achieve higher growth performance and yield as well as more resistance to biotic and abiotic stresses. In SRI rice fields, many and diverse microbes exist and interact with rice plants. Subsequently, this synergetic relationships between SRI methods and microbes affected the rice plant growth, physiological processes, yield, and patterns of gene expression.

Numerous studies have reported numerous plant-beneficial microorganisms such as *Bacillus*, *Trichoderma*, *Aspergillus*, *Penicillium*, *Clostridium*, and *Azotobacter* being more abundant in the rhizospheres of rice under SRI crop management [51,52,53,54]. This is further evidence that management practices have definite effects on the microbiomes of roots. Figure 1 gives an insight into how agricultural management practices such as SRI affect the soil microbial communities and activities.

### 2.2. The Phyllosphere

The aerial parts of rice plants, particularly their leaves, provide an open habitat that is colonized by many diverse microorganisms, many of which contribute to plants’ growth and fitness. The phyllosphere is a dynamic habitat with its resident microbes on or around the leaves subjected to varying environmental conditions such as temperature, water availability, moisture, solar radiation, and elevation [55].

The phyllospheric microbiome population is also much influenced by cultivation methods and chemical fertilizer applications. Rice plants grown under different methods of cultivation such as SRI and conventional methods exhibited different physiological activity and microbial populations, for example. This can be attributed to their respective soil milieus (aerobic vs. anaerobic) and changes in the quantity and forms of introduced nutrients and in the physiochemical properties of the leaves [41,56].

As an open environment that is subject to diverse biotic stimuli, e.g., insect and pathogen invasions, the phyllosphere microbiome is composed of a mixture of commensal, beneficial, and pathogenic species [57]. Roman-Reyna et al. [58] found that the microbial community composition on rice leaves could be delineated in terms of 12 genera that span a range from commensal or pathogenic to beneficial, e.g., *Clostridium*, *Bacillus*, *Helicobacter*, *Azotobacter,* and *Pseudomonas.*

Overall, biotic and abiotic stimuli as well as anthropogenic influences, e.g., cultivation methods and fertilizer application, played important roles in shaping both the structural (taxonomical) and functional attributes of phyllosphere microbiomes. These factors make the rice phyllosphere a dynamic and heterogeneous environment.

### 2.3. The Endosphere

This refers to the plants’ internal domain, which is inhabited by endophytes, both bacteria or fungi, that live most of their life cycle inside plant tissues without causing pathogenic symptoms [59,60]. As integral parts of plants’ structure and functioning, endophytic microorganisms are known to improve plant health, performance, and adaptation to both biotic and abiotic stresses [11,61].

Microorganisms in the endosphere can vary over time. Siderophore-producing bacteria belonging to the genera *Sphingomonas*, *Pseudomonas*, *Burkholderia*, and *Enterobacter* are primarily detected in rice plant tissues during plants’ vegetative stage, when the plants benefit most from siderophores, giving them greater access to iron and other elements in the soil. The endophytic bacterial genus *Pantoea*, on the other hand, is predominant in roots at the time of tillering and then in the leaves at subsequent stages [62]. 

There can also be locational variations in the inhabitation of plant endospheres. In China, for example, the structure of microbial communities within root endospheres was found to differ significantly between the municipalities of Taoyuan, an ecosite with ultrahigh rice yield, and Jinghongdi. Thirteen phyla, primarily *Verrucomicrobia*, *Proteobacteria*, *Planctomycetes,* and *Bacteroidetes*, were identified between the two sites. Metagenomic analysis showed that Taoyuan had more taxa, e.g., *Thaumarchaeota* and *Nitrospira*, which had nitrogen metabolism functions that correlated with mechanisms for the very high yield [63].

In addition to colonizing plant organs, endophytic bacteria are also associated with the seeds (grains) of rice plants. Bacteria genera within the classes Alpha- and Gamma-proteobacteria, Flavobacteria, Bacilli, and Actinobacteria have been observed to reside within rice seeds [64]. Microbial interactions occurring on, around, and inside seeds are important for subsequent plant fitness, since seed-borne microorganisms are the initial source of inoculum for the plant microbiome and are important for early plant development and early plant vigor [65,66].

## 3. Microbial Contributions for Promoting Rice Growth and Yield: From Earlier Views to the Omics Era

The use of microorganisms to enhance rice growth and production is now regarded as an eco-friendly way to carry out intensified agriculture without degrading the environment with agrochemical products or mechanical interventions. Over recent decades, interest in the application of microorganisms in rice production has increased rapidly due to their ability to act as plant-growth regulators [17].

Already in the 1970s and 1980s, various studies conducted on the association of N_2_-fixing bacteria with rice plants indicated that bacterial inoculation could improve plant growth and rice yield [67,68]. However, as rice plants are not leguminous, the benefits were not from N-fixation in nodules formed on plant roots. The inoculation of rice seedlings with *Azospirillum* promoted early tillering and the better reproductive performance of rice plants. It was found to significantly increase the grain-filling rate and the grain weight per plant at harvest time [69].

In the 1990s, biological N_2_ fixation was seen as the most effective system for sustaining production in low-input, traditional rice cultivation [70]. In Egypt, it was found that the association of *Rhizobium leguminosarum* with rice plants could significantly increase the shoot and root growth of these plants, their grain yield, and their nitrogen-use efficiency [71]. This was somewhat surprising, because such effects had previously been observed with legumes, not grass-family (*Poaceae* or *Gramineae*) plants which include rice, wheat, and most cereal crops.

Further studies on the effects of *Rhizobium* on rice plants were conducted in the 2000s. The growth and yield responses of rice plants to inoculation with two bacterial strains (*R. leguminosarum* E11 and *Rhizobium* sp. IRBG74) showed significantly increased grain and higher straw yields at the maturity stage [72]. A multinational collaborative study with three field experiments in Egypt reported that the *Rhizobium* inoculation of rice plants significantly increased their biomass, nutrient uptake (due to more robust root architecture), grain yield, fertilizer efficiency, harvest index, and grain nutritional value. Further experiments of selected rhizobial endophytes on rice showed that they produced cell-bound cellulase and polygalacturonase enzymes that hydrolyze glycosidic bonds in plant cell walls plus certain bacteriocins that can inhibit the growth of undesirable microbes [73].

Chi et al. [74] examined the infection, dissemination, and colonization patterns of rhizobial bacteria within plant tissues following seedling inoculation with one of five selected species, observing their effects on the same variety of rice grown in the same soil. The different species of rhizobia were tagged with green fluorescent protein (*gfp*) markers for better ascertaining their influence on agronomic characteristics of rice. The five sets of seedlings respectively inoculated with different bacterial species were raised under the same greenhouse conditions; a sixth set of plants inoculated with dead bacteria was grown alongside them as a control.

This research showed a dynamic process of infection that started with the rhizobia surface colonizing the roots’ rhizoplane. This was followed by the endophytic colonization of rice root tissues and then by the rhizobia endophytically ascending into the stem, leaf sheathes, and leaves of the plants, where their populations reached as high as 9 × 10^10^ rhizobia per cm^3^ of infected (inhabited) rice tissue. The rice plants that had been infected with living rhizobia, compared to plants inoculated by dead rhizobia of the same type, produced significantly higher root and shoot biomass with greater rates of photosynthesis, more stomatal conductance, greater transpiration velocity, water-use efficiency, and flag leaf area, plus higher measured levels of phytohormones that stimulate and regulate growth. 

A study using strain *Azospirillum* sp. B510 to evaluate its efficacy with different rice cultivars under field conditions showed that the growth of rice plants, especially tiller number at the early growth stage, varied considerably according to rice genotype, as well as the level of soil nitrogen. So, the plant genotype (cultivar) needs to be considered along with the timing of bacterial inoculation and soil and nutrient management practices when applying *Azospirillum*-based biofertilizer as a soil amendment [75]. 

Research in Madagascar in 2000 and 2001 on the effects of using SRI vs. conventional crop management on rice plant tillering and yield, considering also the population density of the bacterium *Azospirillum* inhabiting the plant roots under both cultivation systems, indicated a very large increase in the numbers of endophytic *Azospirillum* spp. associated with SRI management, with a doubling or tripling of grain yield depending on soil quality. Under SRI crop management, the populations of *Azospirillum* residing in the roots of SRI rice plants were many times greater than were counted in the roots of rice plants grown conventionally under the same soil and climatic conditions (reported in Uphoff et al. [76]).

In recent years, microbial consortia have been also developed for improving rice plant productivity, compared with applying single-strain, microbe-based inoculants [77]. Jha et al. [78] have developed a microbial consortium that consisted of *Pseudomonas*, *Azospirillum,* and cyanobacteria for improving rice growth. This consortium increased rice plants’ micro- and macronutrient uptake, their growth, and grain yield. Prasanna et al. [79] evaluated the capability of three selected microbial strains (*Providencia* sp. PR3, *Brevundimonas* sp. PR7, and *Ochrobacterium* sp. PR10) as well as three strains of cyanobacteria (*Anabaena* sp. CR1, *Calothrix* sp. CR2, and *Anabaena* sp. CR3) and also combinations of them to assess their beneficial effects, if any, on rice growth and yield. The microbial consortia significantly enhanced rice growth and grain yield and improved soil health, making it possible to reduce the application of inorganic nitrogen fertilizer by 40–80 kg ha^−1^ without sacrificing grain yield.

In recent years, many studies have used multi-omics approaches to elucidate microbial involvement in the promotion of growth and yield of rice plants [80]. A comparative proteomic study done in China [81] indicated that compared to controls, the functional protein profiles in three categories of rice plant tissues (in the roots, leaf sheaths, and leaves) were differentially up-regulated or down-regulated after inoculation with bacterial strain *Sinorhizobium meliloti* 1021.

Proteins related to photosynthesis capability were up-regulated in the leaf sheath and leaves, while the proteins up-regulated in the roots were mostly linked to plant defense mechanisms. The study also demonstrated an increase in the transport proteins linked to the reaction of chloroplasts to light and dark as well as to the efficient distribution of nutrients within the plant. These enhancements at the cellular level contributed to a higher rate of photosynthesis within the rice plant leaves.

In a subsequent set of trials using transcriptomic analysis [82], these researchers evaluated the effects on plants’ gene expression of inoculating rice seedlings with *S. meliloti* 1021. Their analysis identified more than 2400 differentially-expressed genes (DEGs) in rhizobially-inoculated rice plants vis-à-vis control plants. The genes that were up- or down-regulated in the presence of microbial inoculants were ones known to be involved in processes such as phytohormone production, photosynthetic efficiency, carbohydrate metabolism, cell division, and wall expansion. These effects were induced in rice seedling shoots after the roots had been colonized by *S. meliloti* 1021, so there was a systemic rather than localized response.

These researchers proposed a model to account for how rice seedling roots interact with the *S. meliloti* 1021 around and on them. First, *S. meliloti* 1021 secretes some bioactive signaling chemicals that are recognizable to rice receptor proteins (PRRs, FLS2, and LRR-RLKs). These bioactive signals attach to rice root cells around the area of bacterial colonization and primary infection. After recognizing and transducing these bacterial signals, DEGs that are involved in the production of phytohormones regulating plant growth and development such as auxins, gibberellins, and cytokinins are altered in the rice seedling shoots, and then, the bacteria ascend within the plant into more rice tissues where they live symbiotically, still affecting plant gene expression.

The recognition process between bacterial and plant cells subsequently modulates some plant genes that are involved in the regulation of cell cycles such as *CycA*, *CycB*, *CycD1*, *D2,* and *D3*, impacting and accelerating cell division. These cellular changes also enhance several other processes that promote plant growth and development, photosynthesis capacity, C and N metabolisms, phytohormone production, and other agronomic characteristics [82].

Tang et al. [83] have documented accelerated plant growth and yield enhancement as well as improvement in the quality and mineral nutrition of rice grains after rice plant inoculation with the endophytic fungus *Phomopsis liquidambaris*. Further transcriptomic analysis showed that genes related to certain transporter genes for N (*OsAMT1;4* and *OsNRT1;1*) and for P (*OsPT1* and *OsPht1;2*) were up-regulated during the seedling stage after inoculation with *P. liquidambaris*.

The same results were obtained during the heading stage when rice plants were inoculated with *P. liquidambaris*. This affected the up-regulation of transporter genes in rice roots for P, Zn, and Fe (*OsPT1*, *OsPht1;2*, *OsZIP3*, *OsZIP4*, *OsIRT1,* and *OsIRT2*) and for N, P, Zn, and Fe (*OsAMT1;4*, *OsPht1*, *OsZIP4,* and *OsIRT1*). Significant alteration was also recorded for genes involved in Zn and Fe transport in rice roots, such as *OsZIP3* and *OsIRT2*, and in the transport of P, Zn, and Fe during the ripening stage (*OsPht1;2*, *OsZIP3*, *OsZIP4,* and *OsIRT1*).

Fungi belonging to the genus *Trichoderma* have been frequently reported to be able to colonize rice roots endophytically [84,85]. For example, a study in Nepal revealed that the inoculation of rice seedlings with *Trichoderma* significantly increased plant growth and grain yield compared to that of rice plants in adjacent untreated plots [86]. 

A novel isolate growth-promoting fungus, *T. asperellum* SL2, has been shown to enhance the rice germination, seedling and vegetative growth, plant vigor, grain yield, photosynthetic rate, stomatal conductance, and water-use efficiency of rice plants [87,88]. Transcriptomic analysis revealed that many genes linked to molecular processes at the cell level in rice plants were significantly up-regulated when inoculated with *T. asperellum* SL2. These included genes relating to the synthesis of rubisco (*RBCS*, *OsRBCS1,* and *OsRBCS2*); others involved in stress tolerance (*CYP38* and *CYP20-2*); a gene linked to gibberellin regulation (*OsGAE1*); a gene related to rice tillering (*MOC1*); and a gene related to the uptake of phosphorus (*OsPHR2*) [87,88,89].

In addition to being able to modulate biochemical and molecular signals within rice cells, microbes are known to be able to provide a range of services and benefits to the rice plant through a variety of mechanisms, including organic matter mineralization, biological nitrogen fixation, P-solubilization, Fe-chelation, and the secretion of phytohormones such as indole acetic acid, gibberellins, and cytokinins [23,34]. Documentation regarding the contributions of microorganisms toward the enhancement of rice plant yield and grain production is summarized in Table 1.

## 4. Services of Microbes in Protecting Rice Plants against Plant Diseases

**Figure 2:**
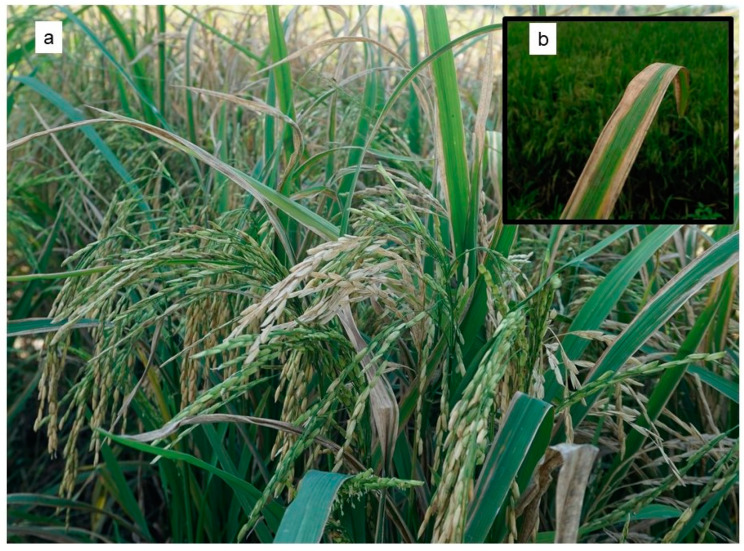

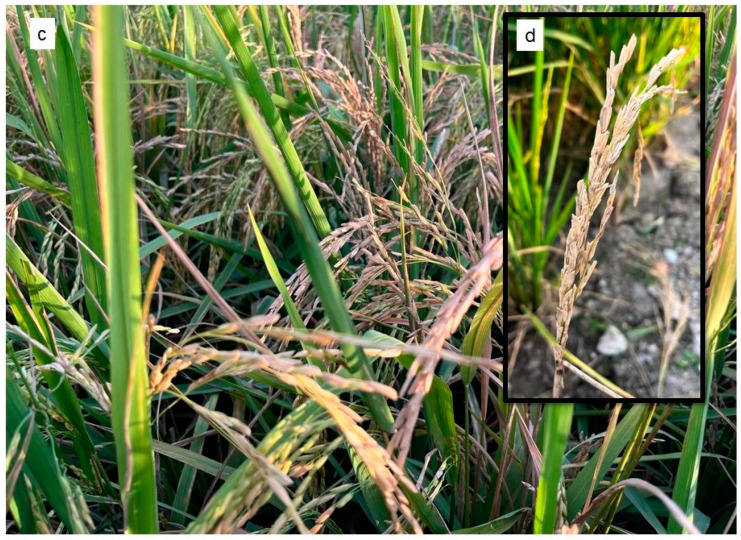
Excessive use of biocides and chemical fertilizers resulted in the severe outbreaks of various rice diseases in Selangor, Malaysia: (**a**) Rice plants infected by *Pantoea* spp. with a yellowish leaf blight disease lesion on the leaves that leads to unfilled, empty, and discolored grains (photo courtesy of Muhammad Nazri Ishak), (**b**) Close-up view of the infected leaf with a lesion at the edge (adapted from Doni et al. [16], the journal does not require permission to use materials), (**c**) Typical field symptoms of bacterial panicle blight caused by *Burkholderia glumae* with discoloration and sterility of grain as well as rotting and panicle blanking, (**d**) A close look at the severely infected panicle. The panicle remains upright rather than bending down with the weight of the grain (photo courtesy of Nurul Shamsinah Mohd Suhaimi). Therefore, the use of beneficial microorganisms for the biological control of plant diseases needs to be encouraged and promoted as a powerful solution to replace toxic chemical biocides.

At present, plant diseases are controlled mostly by using chemical biocides and in some cases by cultural practices. However, the widespread use of agrochemical products in agriculture has been a subject of public concern and scrutiny due to potential harmful effects on the environment, undesirable effects on non-target organisms, and the possible carcinogenicity of some chemical elements [7]. Plus, the intensive use of biocides has led to the development of resistance applications leading to outbreaks of rice diseases (Figure 2). An alternative method for controlling plant diseases is by strengthening plants’ own immunity to diseases through plant–microbial interaction [104]. Several microorganisms belonging to different genera known to elicit induced systemic resistance (ISR) in plants are reported to be effective tools for the biological control of certain plant pathogens [105].

### 4.1. Fungal Diseases

*Trichoderma* spp. are widely used as biocontrol agents for major fungal rice diseases such sheath blight [106,107], rice blast [108], and rice brown spot [109]. The mechanisms of *Trichoderma* spp. for protecting rice plants from pathogenic fungi are several, i.e., competition and rhizosphere competence; mycoparasitism and antibiotic production; antibiosis; degradation of toxins produced by pathogens; production of cell wall-degrading enzymes; inactivation of pathogens’ enzymatic pathways; and induction of defense responses in host plants [110,111]. Transcriptomic analysis has revealed that at least 18 genes related to plant-defense responses are significantly up-regulated after *T. asperellum* SL2 inoculation in rice plants. Furthermore, *T. asperellum* SL2 is also able to induce systemic acquired resistance (SAR) in rice plants by reprogramming the molecular signaling of plant cells [88].

*Pseudomonas* spp. have been studied for decades as model microorganisms for biological control, and they have proved to be effective biocontrol agents for resisting various soil-borne plant diseases [112,113]. It has been known for some time that *P. fluorescens* inhibits the mycelial growth of the sheath blight fungus *R. solani* and increases chitinase and peroxidase activity in rice, thereby inducing systemic resistance against *R. solani* [114]. A study by Patel et al. [115] indicates that *Pseudomonas* produces a volatile organic compound (pyrazine) that can suppress infection by *Magnoporthe oryzae* in rice. A talc-based bioformulation of *P. fluorescens* has also shown biocontrol potential against sheath blight in rice. In field trials, it was found that this microorganism could reduce the incidence of sheath blight in rice by up to 62%, with 12–21% more grain yield [116].

Another prominent genus used for biocontrol, *Bacillus*, can inhibit the spread of major rice diseases such as sheath blight and blast. Antifungal compounds produced by *B. subtilis* show a strong synergistic inhibitory effect on the hyphal growth of *Pyricularia grisea* and *R. solani*, the respective causative agents for blast and sheath blight in rice [117]. 

Another study that employed a strain of endophytic bacterium *Bacillus* sp. EBPBS4 for managing sheath blight showed it to be antagonistic toward the fungal pathogen *R. solani* [118].

Subsequently, a proteomic study indicated that in rice plants treated with this strain of *Bacillus*, there was an up-regulation of putative disease-resistance proteins such as RGA1, NBS-LRR, serine threonine protein kinase, chitinase, β 1–3 glucanase, ascorbate peroxidases, hydroxyl methyl CoA ligase, PAL, and iron superoxide dismutase. The up-regulation of these defense proteins in plants treated with *Bacillus* sp. EBPBS4 was what apparently led to its protective effect against *R. solani* [118].

All of the studies discussed above highlighted that microorganisms could be applied as effective biocontrol agents for more profitable and sustainable rice crop production, especially for controlling fungal diseases while enhancing rice production. Further information on the capability of microbiomes to protect rice plants against different fungal pathogens is summarized in Table 2.

### 4.2. Bacterial Diseases

Leaf blight disease caused by *Xanthomonas oryzae* pv. *oryzae* and *Pantoea* spp. is a major disease faced by rice growers in many countries, causing significant economic losses in rice yield world-wide, since the yield loss due to leaf blight can be more than 70% [16,130]. Over the years, many microbial inoculants have been evaluated as potential biocontrol agents against various bacterial diseases. For example, *Burkholderia amyloliquefaciens* has been shown to possess biocontrol capacity against *X. oryzae* pv. *oryzae* and *X*. *oryzae* pv. *oryzicola*, which are the agents respectively causing leaf blight and bacterial leaf streak in rice. In this case of *B. amyloliquefaciens*, we know that this bacterium produces two antibiotic compounds (difficidin and bacilysin) that down-regulate the expression of genes affecting *Xanthomonas* virulence, cell division, and protein and cell wall synthesis [131].

The combined application of *T. harzianum* and *P. fluorescens* has shown a strong antagonistic effect against *X. oryzae* pv. *oryzae*. The inoculation of *T. harzianum* and *P. fluorescens* synergistically reduced the incidence of leaf blight in inoculated plants compared to the untreated plant controls. Several parameters of rice plant growth and yield were also increased by the combined application of *T. harzianum* and *P. fluorescens*. The protection against *X. oryzae* pv. *oryzae* provided by this fungal–bacterial combination is due to an increase in the lignification of cell walls and in the activities of peroxidase, phenylalanine ammonia-lyase, and 4-coumarate-CoA ligase enzymes in rice leaves [132,133].

Another important bacterial disease in rice is bacterial panicle blight caused by *B. glumae.* Several *Bacillus* spp. significantly suppress the development of bacterial panicle blight caused by *B. glumae* under field conditions [134]. In recent trials, *Bacillus* spp. showed a biocontrol capacity in the greenhouse for reducing *B. glumae* infection by as much as 63.5%, while the populations of *B. glumae* in the plant were diminished by at least two orders of magnitude [135].

The potency of harnessing beneficial microbiomes to control bacterial diseases in rice production has become more apparent every year. Table 3 summarizes the use of microbial inoculants for the biological control of a variety of bacterial pathogens in rice.

## 5. Microbial Involvement in Conferring Abiotic Stress Tolerance in Rice

In addition to protecting against various biotic stresses, plant microbiomes also support plants’ resilience against abiotic stresses including drought, salinity, and heavy metals in the soil. Over the years, some microbes, especially fungal endophytes, have come to be widely regarded as important agents that can confer plant stress through plant–microbe symbiosis [142].

### 5.1. Drought Stress

There are numerous reports of fungal symbionts conferring drought-stress resistance on host plants. *T. harzianum* significantly increases the ability of rice plants to tolerate drought stress with the explanation that this fungus manipulates stomatal conductance, leaf greenness, and net photosynthesis, decreasing proline, malondialdehyde, and H_2_O_2_ contents, and it increases superoxide dismutase levels, plant height, total dry matter, and relative chlorophyll content in rice plants [143,144]. The research indicated that a dose of *T. harzianum* inoculant (30 g/L) is an effective means for improving drought tolerance in rice [144].

Key genes related to metabolic pathways that are affected by inoculation with *T. asperellum* include phenylpropanoid (*PAL*), superoxide dismutation (*SODs*), H_2_O_2_ peroxidation (*APX, PO*), and oxidative defense response (*CAT*). Under drought conditions, these were over-expressed in rice plants after inoculation with *T. asperellum*. Enhancement of the expression of drought-adaptation genes such as *OSPiP* and *DHN* is also reported as a defense mechanism activated by *T. asperellum* against drought stress [145].

Transcriptomic analysis of drought-challenged rice plants bioprimed by *T. harzianum* showed substantial alterations in gene expression compared to untreated control plants [146]. Out of the 2506 DEGs identified by next-generation sequencing analysis, 382 were exclusively expressed in plants inoculated with *T. harzianum*. Comparative analysis of up-regulated and down-regulated genes under drought conditions showed that 1053 genes were up-regulated and 733 genes were down-regulated in the *T. harzianum*-inoculated plants. The genes that were exclusively expressed or up-regulated in such plants were mostly genes related to photosynthetic and antioxidative capacities, e.g., plastocyanin, small units of rubisco, PSI subunit Q, PSII subunit PSBY, osmoproteins, proline-rich protein, aquaporins, stress-enhanced proteins, and chaperonins. Hence, biopriming with *T. harzianum* was seen to aid rice plants in a multifaceted, simultaneous manner that resulted to enhanced drought-stress tolerance.

*P. fluorescens* has also been reported as capable of increasing rice plants’ resistance to drought stress by increasing the activity of protective enzymes, including β-1,3-glucanase, guaiacol peroxidase, peroxidase, phenylalanine ammonia lyase, and superoxide dismutase [147]. A real-time qPCR analysis showed that six genes known to be related to plants’ stress tolerance (*COX1*, *PKDP*, *bZIP1*, *AP2-EREBP*, *Hsp20,* and *COC1*) were up-regulated in drought-stressed rice plants after inoculation with *P. fluorescens* [148].

*B. amyloliquefaciens* is also known to have a positive effect for protecting rice plants from drought stress by modulating various biochemical and physiological activities in the plants, such as membrane integrity and the gene expression of drought-related genes [149]. In addition, AMF were also reported to be effective in enhancing the ability of rice plants to cope with drought stress by decreasing the shoot water potential content of an antioxidant, glutathione [150]. All these results have showed a remarkable influence from microbial colonization, which appears to be a key element for inducing systemic drought-tolerance.

### 5.2. Salinity Stress

One approach to solve the constraint of salt stress is by interposing beneficial microbes [151]. Fungal endophytes have been reported to confer beneficial effects for salt-stressed plants by various direct and indirect mechanisms [152]. *T. harzianum* has the ability to increase rice plants’ resistance to salinity, as under saline conditions, plants treated with this symbiotic fungus showed higher relative water content and more stomatal conductance, a greater rate of photosynthesis, and higher pigment and proline concentrations compared to untreated plants [153,154].

*B. pumilus* has conferred salt-stress tolerance in rice plants by enhancing their antioxidant enzyme activities, such as catalase by 15.1–32.9% and superoxide dismutase by 8.7–26.6%. *B. pumilus* also played a role in accelerating the activities of certain soil enzymes such as alkaline phosphatase (by 18.4–53.5%), acid phosphatase (by 28.4–46%), urease (by 14.8–47.8%), and β-glucosidase (by 25.2–56.1%) in inoculated pots as compared with non-inoculated pots, which neutralized the negative effects of salinity [155].

A fungal endophyte *Piriformospora indica* has been found to regulate salt stress in rice plants by up-regulating certain genes related to salt stress, such as *OsSTK*, *OsLEAP*, *OsAP1*, *OsMPIX*, *Os40S27*, *OsSIP*, and *OsSOS1* [156]. In a different set of trials, rice seedlings that had been inoculated with the bacterial endophyte *P*. *alhagi* exhibited significantly higher activities of certain antioxidant enzymes (SOD, POD, and CAT) and greater plant biomass and chlorophyll content than did uninoculated seedlings in salt-stress evaluations [157]. These studies indicate a capacity of certain endophytic microbes to counteract salinity stress in rice plants and thereby to improve rice production on saline soil.

### 5.3. Heavy Metal Stress

Microorganisms can also play an effective role in acclimatizing plants to grow in metalliferous environments and to improve plants’ tolerance of heavy metals in their soil environment [158]. Plant-associated microbes can decrease the accumulation of such metals in plant tissues and can help to reduce the bioavailability of metals in the soil through various mechanisms [159]. A cadmium-tolerant bacterium *Cupriavidus taiwanensis* was reported to transform a toxic, soluble CdCl_2_ into a nontoxic, insoluble CdS, and it subsequently decreased the accumulation of cadmium in rice plants by 61% [160]. Moreover, it has been seen that rice plants treated with *Trichoderma* spp. had a gain in biomass that appeared correlated with their inducing a lower concentration of the cadmium in these plants that was inhibiting their growth [161].

Another study demonstrated that the heavy-metal-resistant bacteria *Ochrobactrum* spp. and *Bacillus* spp. increased the germination percentage of rice seeds, relative root elongation of seedlings, and the amylase and protease activities in arsenic/cadmium-treated rice plants. The results showed that the activity of superoxide dismutase and the level of malondialdehyde in rice roots were lowered by bacterial inoculation [162]. Recent studies conducted separately by Wang et al. [163] and Pramanik et al. [164] confirmed that *Pseudomonas* spp. significantly reduces cadmium uptake and accumulation in different parts of rice plants. The capacity of *Pseudomonas* spp. to mediate the negative effects of cadmium on rice plants’ performance is closely linked to its affecting changes in soil pH and enzyme activities.

Arbuscular mycorrhiza fungi exert some protective effects against the combined toxicity of copper, zinc, lead, and cadmium in contaminated soil, while at the same time increasing the shoot and root biomass of rice plants [165]. The inoculation of upland rice with AMF also has increased the total phosphorus uptake and decreased arsenic uptake [166].

Bacteria belonging to the genus *Pantoea* have showed a promising capacity to mediate the negative effects of arsenic stress as well as to act as phytostimulants in rice plants. A study conducted by Ghosh et al. [167]. suggests that the inoculation of rice plants with *P. dispersa* significantly improves the morpho-biochemical characteristics of the plants such as increased enzymatic activities and reduced arsenic uptake into rice tissues. All of these results indicate that microbes have promising roles to play in bioremediation as well as in improving plants’ tolerance to heavy-metal stress.

## 6. Conclusions

The continuing increase in global population and the ascendance of environmental threats such as climate change present a strenuous challenge for producing sufficient rice worldwide. To meet global demand, rice yields will need to be increased with diminishing land and water resources per capita.

Current conventional rice farming methods that rely heavily on chemical inputs and varietal improvement have not been increasing the production of rice sufficiently in recent decades, and agrochemical dependence poses serious threats to environmental quality and sustainability. Natural and biological approaches that use beneficial microorganisms in association with other validated agroecological approaches offer untapped opportunities, increasing rice production in eco-friendly ways.

**Figure 3:**
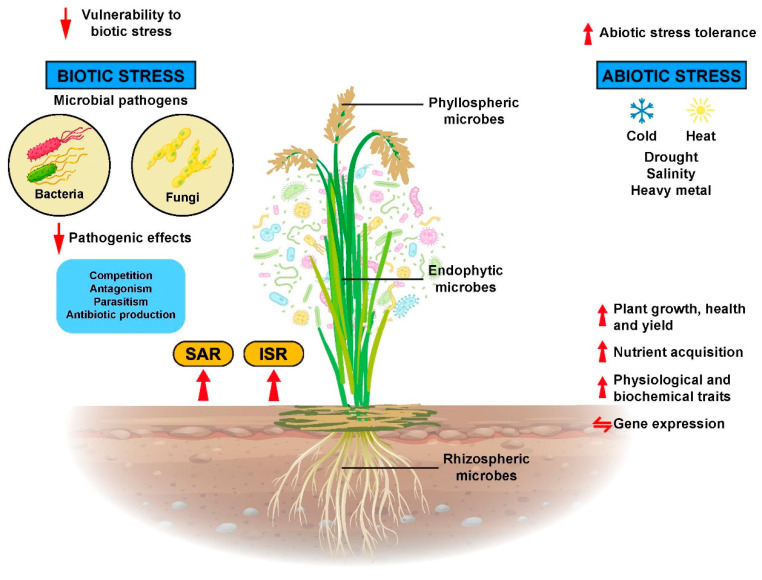
Microbes that are residing in, on, and inside the rice plants affected rice plant growth, physiological, biochemical, and molecular processes. Thereby, they increase rice plant growth, development, and yield as well as more resistance to biotic and abiotic stresses.

In their association with rice plants, microorganisms can use the plants as habitat or just colonize the rice roots or plant surfaces. Once established, microorganisms release bioactive signals that are recognizable by the rice plant cell receptors, and they subsequently enhance plant growth, confer tolerance to abiotic stress, increase disease resistance, alter patterns of gene expression in beneficial ways, improve physiological and biochemical traits, and enhance the nutrient uptake of rice plants (Figure 3).

However, still, much investigation remains to be done to better understand rice plant–microbe interactions, especially with regard to: (i) microbiome cross-talk interaction with rice plants; (ii) the isolation and characterization of more compatible strains of microbes that can be utilized in cost-effective ways for commercialization; (iii) understanding the complex composition of the microbiome in the soil, linking microbiome composition with functions; and (iv) understanding of microbial–rice plant associations and their involved mechanisms using omics-based approaches such as proteomics, transcriptomics, metagenomics, and metabolomics.

## Figures and Tables

**Table 1:** Contributions of microorganisms for the enhancement of rice yield.

Microbes	Experimental Set-Up	Growth Enhancement Effects	Yield Increment (%)	References
*Beijerinckia indica*	Field	Enhanced number of tillers and plant height	27–29	[90]
*R. leguminosarum* E11; *Rhizobium* sp. IRBG74; and *Bradyrhizobium* sp. IRBG271	Pot	Enhanced shoot biomass and NPK uptake	8–22	[91]
Consortium of *Azotobacter, Bacillus, Enterobacter,* and *Xanthobacter*	Pot	Enhanced shoot biomass and N uptake	15–18	[92]
*Trichoderma* spp.	Field	Enhanced number of tillers, plant height, and biomass	18	[93]
Consortium of *Anabaena variabilis, Tolypothrix tenuis, Nostoc muscorum,* and *Aulosira fertilissima*	Field	Enhanced grain yield	37.97	[94]
*Azotobacter* spp.	Field	Enhanced N uptake	67–93	[95]
*Azospirillum* sp. B510	Field	Enhanced number of tillers and plant height	17	[96]
*P. fluorescens*	Field	Enhanced number of tillers, plant height, and biomass	20.6–26.9	[97]
Consortium of *Providencia* sp., *Brevundimonas* sp., and *Ochrobacterium* sp.	Pot	Enhanced plant biomass	19.02	[79]
*T. viride*	Field	Enhanced number of tillers, plant height, and biomass	31	[86]
Consortium of *Pseudomonas* spp., *Azotobacter chroococcum*, and *Azospirillum brasilense*	Pot	Enhanced shoot biomass and NPK uptake	15.3	[98]
*Cellulomonas flavigena*	Pot	Enhanced number of tillers and plant height	18	[99]
*T. asperellum* SL2	Field	Enhanced plant height, biomass, number of tillers, photosynthetic rate, water-use efficiency, and gene expression	30	[100]
*Serratia* spp.	Field	Enhanced stomatal conductance, nutrient uptake, shoot dry matter, number of grains per plant, and grain yield	7–22	[101]
Consortium of *B. ubonensis* la3c3, *B. vietnamiensis* la1a4, and *Citrobacter bitternis* p9a3m	Field	Enhanced NPK uptake	2.5–13.5	[102]
Consortium of *Pseudomonas* sp. and *Penicillium* sp.	Field	Enhanced P uptake	16	[103]

**Table 2:** Microbes and their biocontrol regulation activities against fungal diseases in rice.

Microbes	Phytopathogens	Observed Effects	References
*P. fluorescens*	*P. oryzae*	Triggered ISR in rice against *P. oryzae*	[119]
*Serratia marcescens*	*R. solani*	Reduced the incidence of sheath blight	[120]
*Bacillus subtilis*	*R. solani*	Reduced disease incidence and severity of sheath blight	[121]
*Glomus intraradices*	*M. oryzae*	Up-regulation of defense-response genes such as *OsNPR1*, *OsAP2*, *OsEREBP* and *OsJAmyb*	[122]
*B. oryzicola*	*Gibberella fujikuroi*	Reduced *bakanae* severity by 46–78%	[123]
*Streptomyces* spp.	*M. oryzae*	Acceleration of defense enzyme activities such as synthesis of catalase, phenylalanine ammonia-lyase, and β-1,3-glucanase	[124]
*P. fluorescens*	*M. oryzae*	Reduced the physiological damage caused by *M. oryzae*	[9]
*Cladosporium cladosporioides*	*M. oryzae*	Modulation of enzymatic activity, and enhanced expression of defense-related genes such as *JIOsPR10*, *LOX-RLL*, and *PR1b*	[125,126,127]
*B. subtlis*	*M. oryzae*	Reduction in blast disease by >50%	[128]
*Talaromyces* spp.	*R. solani*	Up-regulation of defense-related genes, and acceleration of defense enzyme activities	[129]

**Table 3:** Examples of biocontrol effects of beneficial microbes in protecting rice plants from bacterial diseases.

Microbes	Phytopathogens	Observed effects	References
*B. oryzicola*	*B. glumae*	Induction of systemic resistance and promotion of plant growth	[136]
*B. subtilis; B. amyloliquefaciens;* and *B. methyltrophicus*	*X. oryzae* pv. *oryzae*	Activation of ISR, resulting in enhanced activity of defense-related enzymes	[137]
*P. aeruginosa*	*X. oryzae* pv. *oryzae*	Induction of defense-related enzymes	[138]
*Streptomyces* spp.	*B. glumae*	Inhibited the growth of *B. glumae* and increased plant growth	[139]
*B. amyloliquefaciens* and *Aspergillus pseudoporous*	*X. oryzae* pv. *oryzae*	Up-regulation of defense-related enzymes, and acceleration of the activities of defense-related proteins and total phenols	[140]
Consortium of *S. fimicarius*, *S. laurentii*, *P. putida,* and *Metarhizium anisopliae*	*X*. *oryzae* pv. *oryzae*	Reduced the incidence of leaf blight	[141]

## Data Availability

Not applicable.

## Abbreviations

AMF, arbuscular mycorrhizal fungi; APX PO, H_2_O_2_ peroxidation; CAT, oxidative defense response; DEGs, differentially expressed genes; *gfp*, green fluorescent protein; ISR, induced systemic resistance; PAL, phenylpropanoid; POD, peroxidase; qPCR, quantitative polymerase chain reaction; SAR, systemic acquired resistance; SODs, superoxide dismutation; SRI, System of Rice Intensification.
